# Dendritic cell heterogeneity and its role in connective tissue diseases

**DOI:** 10.1186/s41232-025-00388-z

**Published:** 2025-07-28

**Authors:** Saeko Yamada, Yuichi Suwa, Keishi Fujio

**Affiliations:** Department of Allergy and Rheumatology, Graduate School of Medicine, The University of Tokyo, 7-3-1 Hongo, Bunkyo-ku, Tokyo, 113-8655 Japan

**Keywords:** Dendritic cells, Autoimmune disease, Connective tissue disease, Rheumatoid arthritis, Systemic lupus erythematosus, Idiopathic inflammatory myopathies, Systemic sclerosis, Vasculitis, Pulmonary arterial hypertension

## Abstract

By balancing immunity and tolerance, dendritic cells (DCs) are key regulators of immune responses. Recent studies have highlighted the crucial role of these cells in connective tissue diseases. Conventional DCs (cDCs) and plasmacytoid DCs (pDCs) exhibit distinct contributions to disease progression. In systemic lupus erythematosus and systemic sclerosis, pDCs primarily drive pathogenesis via excessive type I interferon production, whereas in rheumatoid arthritis (RA), cDCs play a major role in promoting autoreactive T cell activation. Emerging DC subsets, such as inflammatory DC3s and LAMP3^+^ DCs, have been implicated in RA synovitis. In vasculitis, tissue-resident vascular DCs appear to regulate localized inflammation. Despite advances in single-cell analysis, the functional roles of specific DC subsets remain underexplored in several autoimmune conditions. Understanding DC heterogeneity and function in disease-specific contexts may lead to novel therapeutic strategies targeting DC-mediated immune dysregulation.

## Background

Recognition of antigens by the innate immune system is considered the initiating event triggering self-reactive innate and adaptive immune responses. Dendritic cells (DCs), discovered by Ralph Steinman in 1973, are the most crucial antigen-presenting cells involved in the regulation of both innate and adaptive immunity and maintenance of immune tolerance. DCs comprise a highly heterogeneous cell population with multiple subsets, including conventional DCs (cDCs), plasmacytoid DCs (pDCs), monocyte-derived DCs (moDCs), and tolerogenic DCs (tolDCs). These DC subsets exhibit distinct transcriptional, metabolic, phenotypic, and functional characteristics and play diverse roles in regulating the balance between immunity and tolerance.

In human peripheral blood, the predominant DC subsets are pDCs, characterized by a CD11c^−^ CD123^+^ phenotype, and cDCs, characterized by a CD11c^+^ CD123^−^ phenotype. pDCs express CD303 (BDCA2) and respond rapidly to viral infections by producing high levels of type I interferons (IFNs) via Toll-like receptor (TLR) 7/9 [1]. In contrast, cDCs are responsible for T cell activation. cDCs are further classified into CD141 (BDCA3)^+^ cDC1s, which activate CD8^+^ T cells by cross-presentation via MHC class I, and CD1c (BDCA1)^+^ cDC2s, which strongly activate CD4^+^ T cells [2, 3].

In 2017, Villani et al. identified the DC subsets DC1-DC6 in the human peripheral blood via single-cell RNA sequencing (scRNA-seq) of CD3^−^ CD19^−^ CD56^−^ CD14^−^ HLA-DR^+^ cells [4]. In addition to the well-known pDC (DC6) and cDC subsets (cDC1 [DC1], cDC2 [DC2]), two DC clusters (DC3 and DC4) with monocytic signatures and a new cluster with a unique expression pattern (DC5) were identified. DC5s, also known as AS-DCs, largely overlap with early-stage cDC precursors (pre-DCs) [5] and are believed to develop into cDC2s [6].

Both DC2s and DC3s express *CD1c*, but particularly, DC3s show higher expression of *FCN*, *S100A8*, and *CD14* compared with DC2s; thus, DC3s are thought to resemble moDCs, which are induced in vitro from CD14^+^ monocytes [7]. Some DC3s are inflammatory DC3s (iDC3s), characterized by CD14^+^ CD163^+^, and closely resemble previously reported inflammatory DCs [6]. iDC3s in such environments may polarize toward Th1 and Th17 cells [8]. DC3s exhibit functional characteristics distinct from both cDC2s and monocytes, while more closely resembling those of cDC2s.

While cDCs can activate naïve T cells, tolDCs, which are induced by anti-inflammatory signals or factors that inhibit maturation, exhibit distinct characteristics such as limited maturation, reduced secretion of pro-inflammatory cytokines, increased expression of inhibitory molecules (e.g., IL-10), and promotion of regulatory T cell (Treg) function. TolDCs aim to maintain peripheral immune tolerance and are being explored for clinical application in autoimmune diseases.

Autoimmune rheumatic diseases encompass a complex group of disorders characterized by chronic inflammation and tissue damage. Given that DCs are involved in the initiation and formation of both innate and adaptive immune responses, they are believed to play a pivotal role in the pathogenesis of autoimmune diseases. This review will discuss the role of DCs, including their altered abundances and functions, in major connective tissue diseases.

### The role of DCs in connective tissue diseases

#### Rheumatoid arthritis

Rheumatoid arthritis (RA) is a common autoimmune chronic inflammatory joint disease. Genome-wide association studies (GWAS) have identified *HLA-DRB1* as the most significant genetic factor associated with RA susceptibility [9]. Antigen-presenting cells, including DCs, take up citrullinated proteins, which serve as antigens, and present the proteins via human leukocyte antigen (HLA) molecules, thereby activating CD4^+^ T cells. This process is considered the initial pathogenic event in RA, highlighting the critical role of DCs in disease onset.

While the cDC2 subset has long been considered pathogenic in RA [10–12], recent studies have gradually elucidated the functions of various DC subsets in RA pathogenesis. The changes in DC populations in the peripheral blood of RA patients remain controversial. One study reported an increased population of cDCs, with no change in pDCs in RA peripheral blood [13], whereas several other studies showed decreased populations of both circulating cDCs and pDCs [14–17]. Notably, cDCs have been correlated with RA disease activity, with reduced peripheral cDC numbers in highly active cases [16] and increased numbers in response to tumor necrosis factor (TNF) inhibitor therapy [14]. In cases resistant to treatment, an increased population of pre-DCs has been observed in peripheral blood before treatment initiation [5].

In the synovium of RA patients, CD14^+^ DCs localize around blood vessels [18], and both cDC1 and cDC2 populations are increased [19, 20]. In the synovium, cDC1s exhibit greater T cell-activating capacity compared with their peripheral blood counterparts [19], while cDC2s show upregulated expression of co-stimulatory markers such as CD80 and CD83, as well as CCR7, which promotes local T cell infiltration [20]. These findings suggest that the migration of cDCs from peripheral blood to the joints, along with their differentiation and functional changes, may be associated with RA pathogenesis, disease activity, and treatment responsiveness.

LAMP3^+^ DCs are not observed in peripheral blood but have been detected in the synovium [21, 22]. These cells are also found in tumor tissues, where they exhibit high expression of immunoregulatory molecules and possess the ability to activate lymphocytes [23]. Synovial LAMP3^+^ DCs express CD1c and CCR7, suggesting that they have differentiated from DC2s [23]. Similar to DC2s, they were expanded in the sublining lymphoid niche of active RA synovium and were colocalized with naive T cells [24]. An interesting finding from transcriptomic analyses of synovial data in a large U.S. cohort study, the Accelerating Medicines Partnership (AMP) program, revealed that a DC-enriched phenotype in RA is correlated with increased T cell numbers and is associated with both the synovial aggregate score and density score [22].

As mentioned earlier, recent analyses have highlighted similarities between inflammatory DCs and DC3s [6]. In active RA synovium, inflammatory DC3s have been reported to expand and colocalize with inflammatory T cells, such as CCL5^+^ T peripheral helper (Tph) cells and CCL5^+^ effector memory T (Tem) cells, contributing to their activation [24].

On the other hand, pDCs may play a suppressive role in RA progression. For instance, pDC depletion in mice exacerbates arthritis severity and inflammatory responses [25], although studies in humans remain limited.

In summary, cDCs likely migrate from the peripheral blood to the synovium and undergo functional changes in RA. Within the synovium, functionally mature DC1s and DC2s coexist with newly identified unique subpopulations, including LAMP3^+^ DCs and iDC3s. LAMP3^+^ DCs may activate lymphocytes locally, while iDC3s may drive specific T cell polarization, both of which could be associated with RA pathogenesis and disease activity. Several biologic agents targeting DC–T cell interactions and cytokines produced by DCs are currently used in clinical practice. In addition, Janus kinase inhibitors—such as tofacitinib, baricitinib, and ruxolitinib—suppress DC-mediated T cell activation and can therefore be considered important therapies that indirectly focus on DCs [26–28].

In addition, intra-articular administration of autologous tolDCs in patients with inflammatory arthritis, including RA, has been suggested to provide local improvement in joint symptoms, although systemic symptom relief has not been demonstrated [29]. The safety of intranodal therapy using tolDCs loaded with mB29a, a surrogate autoantigen effective in animal models of arthritis, is currently being evaluated in the TOLERANT trial [30].

### Systemic lupus erythematosus

Systemic lupus erythematosus (SLE) is a complex, multifactorial autoimmune disease that causes severe inflammatory lesions in multiple organs, including the skin, kidneys, joints, cardiovascular system, and nervous system.

#### pDCs in SLE

The IFN signature is considered a characteristic molecular marker of SLE [31], and excessive activation of pDCs along with overproduction of type I IFNs plays a central role in SLE pathogenesis [32, 33]. Indeed, mutations in IFN-related genes contribute to SLE susceptibility and clinical severity [34].

In SLE, the number of circulating pDCs is reduced [35, 36], possibly reflecting the migration of these cells into tissues. As expected, activation of pDCs has been implicated in SLE pathogenesis in multiple studies. In fact, pDCs from SLE patients exhibit enhanced TLR7-dependent IFN-α production, which is correlated with disease activity [37]. pDCs internalize viral or self-derived single-stranded RNA and double-stranded DNA (dsDNA) via the Fcγ receptor IIa, recognize these molecules via innate immune sensors such as TLR7/9, and produce large amounts of type I IFNs in response. Furthermore, production of type I IFNs by pDCs promotes the differentiation of extrafollicular B cells into short-lived antibody-producing cells, enhancing anti-DNA antibody production. This positive feedback mechanism has been observed in Dnase1l3^−/−^ mice [38]. Additionally, in SLE, the level of dsDNA-specific IgE is correlated with disease severity. IgE autoantibodies induce phagocytosis via the high-affinity Fcε receptor I, which then facilitates DNA recognition via TLR9 within phagosomes, significantly enhancing pDC function [39]. Moreover, activated neutrophils in SLE release self-DNA–peptide complexes in the form of neutrophil extracellular traps (NETs), which activate pDCs via TLR9 [40]. The nuclear alarmin interleukin (IL)−33, released during cell death, forms complexes with NETs, accumulates in inflamed tissues, and strongly induces type I IFN production by pDCs in SLE patients in an IL-33 receptor (ST2L)-dependent manner [41].

On the contrary, pDC-mediated induction and functional regulation of other cells also contribute to SLE pathogenesis. One previously highlighted cell type induced by pDCs in response to oxidized mitochondrial DNA-mediated TLR9 activation is CXCR5⁻ CXCR3⁺ PD1^hi^ CD4⁺ T cells (Th10). These cells support B cell activation via IL-10 production and succinate signaling. In patients with proliferative lupus nephritis (LN), this Th10 population is expanded in both peripheral blood and the tubulointerstitial region, suggesting the involvement of these cells in SLE pathogenesis [42]. Interestingly, pDCs also play a role in maintaining immune tolerance by inducing regulatory T cells (Tregs) and suppressing T cell activation [43–46]. Additionally, pDCs promote the differentiation of immature B cells into IL-10-producing CD24⁺CD38^hi^ regulatory B cells (Bregs) via IFN-α secretion and CD40 signaling. In turn, these CD24⁺ CD38^hi^ Bregs suppress type I IFN production by pDCs via IL-10 secretion, forming a regulatory feedback loop. Disruption of this balance is thought to contribute to SLE pathogenesis [47].

Given these mechanisms, therapeutic approaches targeting the activation of pDCs and their regulatory interactions with other immune cells may become a viable strategy for SLE treatment in the future. Specifically, therapeutic strategies under investigation include antibody therapies targeting human pDC-specific surface marker BDCA2, as well as the use of chloroquine to inhibit endosomal activation of TLR7 and TLR9 [48–50]. Inhibitors of Bcl-2, a molecule essential for pDC survival and type I interferon (IFN-I) production, have been shown to induce apoptosis of pDCs and suppress IFN-I production in both lupus patients and mouse models [51]. In addition to anifrolumab, other agents that directly target type I IFNs have demonstrated therapeutic potential; for instance, sifalimumab, a fully human monoclonal antibody, improved disease activity in a phase IIb clinical trial for SLE [52], and long-term administration of antibodies against the IFN-α/β receptor has been shown to ameliorate serological, cellular, and clinical manifestations in murine models [53]. Inhibitors of signaling pathways involved in IFN-I production are also being explored, including Zimlovisertib, an IRAK4 inhibitor that targets a key mediator of IFN-I signaling [54, 55], and agents that suppress the hyperactivation of IRF5, a major regulator of IFN-I production [56].

#### cDCs in SLE

Activated cDCs play a crucial role in immune tolerance breakdown and tissue inflammation. Upon pathogen stimulation, they express co-stimulatory molecules that promote T cell activation and differentiation, as well as enhance B cell activation and autoantibody production in the germinal center (GC). In SLE patients, peripheral blood cDCs exhibit a proinflammatory phenotype and function, characterized by increased differentiation of monocytes into cDCs, upregulated co-stimulatory molecule expression, and elevated IL-8 production, which all contribute to abnormal T cell responses [57, 58]. Single-cell analysis has further elucidated these findings, revealing that inflammatory CD5⁻ CD163⁺ CD14⁺ DCs (iDC3) are increased in the peripheral blood of SLE patients. These cells have a heightened ability to promote Th2 and Th17 cell differentiation and secrete inflammatory mediators, correlating with disease severity [6]. Similarly, single-cell analysis of renal biopsy samples from LN patients has shown that an increase in CD163⁺ DC3 correlates with LN severity [8]. Additionally, in pediatric SLE patients, DC5s are increased in the peripheral blood [59], suggesting the involvement of cDC differentiation and activation in SLE pathogenesis.

One of the hallmarks of autoimmunity in mouse lupus models is the proliferation and differentiation of autoreactive B cells within GCs in lymphoid tissues. Under normal conditions, when the host responds to foreign antigens, follicular dendritic cells (FDCs) maintain GCs by capturing and recirculating immune complexes opsonized by complement. However, in lupus models, FDCs capture and retain self-antigens via CD21, activating type I IFN production in a TLR7/IRF5-dependent manner, thereby promoting GC responses and pathogenic autoantibody production [60]. This suggests that FDCs function as an alternative source of type I IFN, distinct from pDCs, which drive autoimmunity. Since this pathway is conserved in humans, it could represent a potential therapeutic target for SLE.

Furthermore, a specific pathway involving interaction between cDCs and GC B cells has been identified. GC B cells enhance T cell activation by acquiring MHC II-C3 complexes formed on the surface of cDCs via trogocytosis, a process in which they partially engulf the cell membrane [61]. Further investigation into the differentiation pathways of cDCs in SLE and their interactions with T cells, B cells, and other immune cells may lead to a deeper understanding of disease pathogenesis.

In addition to these approaches, the transfer or induction of tolDCs is being considered as a novel therapeutic strategy for treating and ultimately curing SLE [62, 63]. This approach has attracted considerable interest as a means to avoid long-term use of immunosuppressive drugs; however, it has not yet been applied clinically.

### Idiopathic inflammatory myopathy

Idiopathic inflammatory myopathies (IIMs) encompass several heterogeneous phenotypes, including polymyositis (PM), dermatomyositis (DM), and antisynthetase syndrome. For instance, the histological features of DM include CD4^+^ T cell and B cell infiltration, MHC I expression on muscle fibers, and membrane attack complex (MAC) deposition in capillaries. In contrast, PM is characterized by CD8^+^ T cell infiltration [64].

Both pDCs and cDCs have been identified in the muscle tissues of patients with various IIMs, including DM, PM, and inclusion body myositis (IBM). pDCs are predominant in DM, whereas cDCs are more prevalent in PM and IBM [65–67]. Consistent with this, transcriptome analysis of muscle biopsy samples revealed distinct activation patterns of type I IFN-inducible genes across different IIM subtypes, with high expression in DM and low expression in IBM [68]. The distribution patterns of DCs in inflamed muscle tissue mirror the infiltration patterns of T cells, with perivascular localization in DM and endomysial infiltration in PM [69]. Additionally, in DM and PM, the proportion of cDCs in peripheral blood is correlated with the creatine kinase level [70]. In untreated juvenile dermatomyositis, the duration of chronic inflammation appears to be associated with DC maturation and anti-angiogenic vascular remodeling in muscle biopsy samples [71]. These findings suggest that DC accumulation in IIM-affected muscle tissue contributes to the progression of immune-mediated reactions and subsequent muscle damage. In DM, C5b-9 MAC is deposited on capillary endothelial cells, and its activation may promote the migration of immune cells, including CD123^+^ pDCs, into the endomysium [72]. Therefore, one potential mechanism underlying muscle damage in DM involves activation of the complement pathway and DC infiltration.

Co-culture experiments have shown that activated moDCs interact with myoblasts to promote their proliferation and migration, enhance the expression of HLA-ABC, HLA-DR, VLA-5, and VLA-6, and induce muscle damage via inflammatory cytokine secretion [73]. This activation of myoblasts by DCs creates a cytokine-rich environment that may stimulate signaling pathways such as NFκB and MAPK, which in turn influence myoblast differentiation and atrophy [74–76].

In summary, while the pathogenic mechanisms vary across IIM subtypes and remain unclear, therapeutic strategies targeting the IFN pathway and complement system in DM may hold promise.

### Systemic sclerosis

Systemic sclerosis (SSc) is a heterogeneous autoimmune disease of unknown etiology, characterized by fibrosis, vascular abnormalities, and immune dysregulation. In SSc, aberrant distribution and dysfunction of DCs may contribute to the breakdown of immune tolerance and the induction of inflammation. Additionally, recent studies have gradually revealed the direct involvement of DCs in vascular endothelial injury and fibrosis progression, as well as the impact of DCs on disease severity in barrier tissues such as the skin and lungs.

#### pDCs in SSc

Several studies have reported increased levels of pDCs in the skin and lungs, but decreased levels in the peripheral blood, of SSc patients [77–80]. Notably, the severity of lung disease in SSc patients is correlated with the frequency of pDCs in the lungs [80], and depletion of pDCs improved fibrosis in the skin and lungs [77, 80, 81], suggesting that pDCs play a direct role in fibrosis.

Regarding the role of type I IFN in SSc, IFN-α exacerbates skin and lung dysfunction in diffuse cutaneous SSc (dcSSc) [82]. There have also been case reports of patients developing SSc after receiving type I IFN therapy for myeloproliferative disorders or hepatitis C [83, 84]. Conversely, a monoclonal antibody against a type I IFN receptor (IFNAR) subunit 1 receptor anifrolumab has been shown to suppress T cell activation and collagen accumulation, thereby slowing the progression of tissue fibrosis [85]. *IRF7*, which encodes a key regulator of type I IFN signaling, is not only associated with SLE but is also a susceptibility locus for anticentromere autoantibodies in SSc [86]. In SSc patients, IRF7 is upregulated in skin, where it binds to Smad3, a major component of the TGF-β signaling pathway, promoting fibrosis [87]. This suggests that IFN-α contributes to fibrosis progression via IRF7. Additionally, GWAS showed that *IRF8*, a transcription factor essential for pDC development and activation, is associated with limited cutaneous SSc (lcSSc) [88]. Increased expression of miR-618, a microRNA targeting *IRF8*, suppresses pDC development while enhancing IFN-α secretion, thereby contributing to the type I IFN signature in SSc [89]. In interstitial lung disease (ILD) within SSc, lung tissue pDCs show transcriptional activation and upregulation of multiple cellular stress-related pathways, along with increased expression of IFNAR, compared to idiopathic pulmonary fibrosis [90]. Taken together, IRF7, IRF8, and IFNAR represent promising therapeutic targets for SSc.

On the other hand, in the pDCs of SSc patients, chemokine C-X-C motif ligand 4 (CXCL4) plays a central role in inflammation and fibrosis. The CXCL4 level is elevated in the blood and skin of SSc patients [79], in correlation with the presence and progression of complications such as ILD and pulmonary arterial hypertension (PAH) [79, 91]. Specifically, CXCL4 production in pDCs is induced by signaling pathways involving aberrant TLR8 expression and TLR9 activation under hypoxic conditions [77, 92]. pDCs excessively activated by CXCL4 complex with self- or foreign DNA infiltrate tissues and secrete IFN-α [93], contributing to an inflammatory tissue environment. CXCL4 also forms complexes with self-RNA, activating pDCs via TLR7/8, and these complexes are correlated not only with the type I IFN signature but also with the plasma TNF-α level [94, 95]. Furthermore, CXCL4 directly promotes the differentiation of fibroblasts into myofibroblasts, which produce collagen and other extracellular matrix components, thereby exacerbating fibrosis [96]. CXCL4 also enhances monocyte secretion of platelet-derived growth factor (PDGF)-BB, the levels of which are particularly elevated in dcSSc patients, leading to increased deposition of fibronectin and collagen by fibroblasts [97]. In moDCs differentiated in the presence of CXCL4, transcriptomic and epigenetic profiling revealed that CXCL4 dramatically alters monocyte differentiation pathways, inducing a novel pro-inflammatory and pro-fibrotic phenotype [98]. Given its role in the key steps of SSc disease progression, CXCL4 represents a potential therapeutic target.

Several other factors in pDCs may also contribute to SSc pathogenesis. These include reduced expression of RUNX3, a transcription factor involved in DC lineage differentiation [99], abnormalities in the IRE1α/XBP1/PHGDH pathway within the pDC unfolded protein response [100], and metabolic profile alterations [101].

Taken together, pDCs play a central role in the pathogenesis of SSc and represent a promising therapeutic target.

#### cDCs in SSc

Compared with pDCs, cDCs have been investigated less extensively in the pathogenesis of SSc. cDCs from patients with early-stage lcSSc or dcSSc, compared with healthy individuals or patients with long-standing disease, have been shown to produce significantly higher levels of proinflammatory cytokines such as IL-6 and TNF-α in response to TLR-mediated activation, highlighting the potential role of cDCs in the early and progressive phases of SSc [102].

Among the cDC subsets, cDC2s from the peripheral blood of lcSSc patients, compared to healthy individuals, exhibited increased ex vivo production of CXCL10 and higher levels of CXCL8 upon stimulation with lipopolysaccharide and IFN-γ. Meanwhile, cDC2s from dcSSc patients showed increased production of CCL4 compared with healthy individuals [103]. These distinct patterns of inflammatory cytokine production suggest potential differences in the molecular mechanisms underlying the two disease subtypes.

Regarding iDC3s, their proportion in SSc patients did not differ from that in healthy individuals, unlike in SLE [6]. However, the scavenger receptor CD163, which is considered a marker of M2 macrophages, is elevated in the serum of SSc patients [104–106]. Additionally, in SSc patients, the cytokine-producing capacity of cDCs was highly correlated with that of moDCs [102], cells produced from CD14^+^ monocytes after in vitro stimulation [7] and that often share a similar signature with inflammatory DCs [107]. In fact, scRNA-seq analysis of myeloid cells in the skin of some dcSSc patients revealed an increased presence of FCN1⁺ moDCs, which was associated with the severity of skin manifestations [108]. Furthermore, CXCL4, in combination with GM-CSF and IL-4, promotes monocyte differentiation into moDCs [98, 109, 110]. This suggests that under inflammatory conditions and in the presence of CXCL4, monocytes in SSc may differentiate into inflammatory DC3s, contributing to the fibrotic process. Additional studies are needed to further investigate the localization of inflammatory DC3s within affected tissues and their signaling mechanisms across tissue compartments.

Reports on the pathological role of cDCs in SSc remain scarce, and further research is needed to elucidate the role of cDC subsets in disease progression, particularly in relation to different SSc subtypes.

#### cDCs in SSc-PAH

In connective tissue diseases, particularly SSc, pulmonary arterial hypertension can occur as a complication and is referred to as connective tissue disease-associated pulmonary arterial hypertension (CTD-PAH). The number of DCs was found to be increased in SSc-PAH compared with IPAH and healthy controls [111]. In a diagnostic model using an artificial neural network based on signal recognition particle-related pathways, a correlation with activated DCs and CTD-PAH was identified [112], further suggesting a link between DCs and disease pathology.

The number of circulating cDCs is decreased in PAH [113–115]. In CTD-PAH patients, tertiary lymphoid organs (TLOs) have been reported to form around the pulmonary vasculature, where DCs are present [116–120], suggesting the recruitment of DCs to TLOs. TLOs develop in response to prolonged local immune activation and are considered a hallmark of chronic diseases [121]. CCR7 induces DC migration to lymph nodes via lymphatic vessels in response to CCL19 and CCL21. In CCR7-deficient mice, this migration is impaired, leading to the formation of pulmonary TLOs and the development of PAH-like features [122, 123]. Additionally, DCs produce CCL20 and CXCL13, which attract T cells, B cells, and immature DCs.

The mechanisms underlying cDC activation in PAH remain unclear. In SSc, peripheral blood cDC2s produce higher levels of IL-6, IL-10, and TNF-α upon TLR2 and TLR4 stimulation [102, 103]. Similarly, in IPAH and hereditary PAH, the serum levels of IL-6 and IL-10 were found to be elevated and correlated with mortality [124], suggesting that IL-6 and IL-10 may be one of the possible mechanisms underlying SSc-PAH. Furthermore, functional analyses of moDCs and cDCs from SSc patients carrying TLR2 polymorphisms associated with PAH development showed increased IL-6 production in response to TLR2-mediated stimulation [125]. However, in group 1 PAH, including CTD-PAH, treatment with the human IL-6 receptor monoclonal antibody tocilizumab showed limited efficacy [126].

### Vasculitis types other than ANCA-associated vasculitis

Vasculitis (e.g., Takayasu arteritis [TAK], giant cell arteritis [GCA], and polyarteritis nodosa) is characterized by its selective impact on specific vascular beds. However, the molecular mechanisms underlying the induction of vascular wall inflammation in limited anatomical regions remain unclear.

In human arteries, which have a three-layered structure, a distinct population of DCs known as vascular DCs exists within the vessel wall. These DCs localize to the boundary between the adventitia and media, often on the outer side of the lamina elastica externa [127, 128]. Positioned near the neovascularized vasa vasorum in the adventitia, vascular DCs serve as a barrier between the vascular wall access and the immunoprivileged tissue site. An in situ analysis of vascular immunity in steady-state C57BL/6 mice revealed a complete set of DC subsets within the vessel wall. Notably, moDCs, which are generally considered rare under steady-state conditions, accounted for nearly half of the total DC population and thus were the most abundant DC subset [129]. Vascular DCs constitutively express immunosuppressive factors; specifically, cDC1s highly express PD-L1, while moDCs highly express IL-10 (an anti-inflammatory cytokine). However, this immune-tolerant state in vascular tissue is easily disrupted by systemic inflammation [129]. Due to the nature of vascular DCs, detailed investigations into their role in local vascular lesions in human vasculitis patients remain limited.

These DCs, collectively referred to as vascular DCs, have access to pathogen-associated and damage-associated molecular patterns in the bloodstream. Upon TLR stimulation, they break self-tolerance and promote T cell recruitment and activation via expression of co-stimulatory molecules and release of chemokines [130, 131]. Interestingly, the TLR expression profile of vascular DCs varies among different blood vessel types, suggesting that the TLR profile may be involved in the stringent target tissue tropism of inflammatory vasculopathies and protection from immune attacks in different vasculitis diseases affecting distinct blood vessels [132]. For example, TLR2 and TLR4, which are widely expressed in medium- and large-sized blood vessels, are highly expressed in the temporal artery in GCA, where they serve as strong stimulators of DCs [132]. Activation of different TLR subtypes stimulates distinct chemokine pathways. Notably, TLR4-mediated DC stimulation enhances the production of CCL20, leading to the recruitment of CCR6^+^ T cells, which play a key role in vasculitic infiltration. Activated DCs also express chemokines such as CCL18, CCL19, and CCL21, which induce T cell responses, and secrete pro-inflammatory cytokines such as IL-6, thereby inducing an inflammatory environment both within and outside the vasculature. Similarly, in TAK, arterial tissues exhibit high TLR4 expression [133]. TLR4 activation increases the expression of *IL1B*, which encodes the pro-inflammatory molecule IL-1β, as well as *IL1R2*, which encodes the anti-inflammatory decoy receptor IL-1 receptor 2 (IL-1R2), in the peripheral blood mononuclear cells of TAK patients [134]. Notably, high IL-1R2 expression has also been observed in Behçet’s disease (BD), another type of vasculitis that affects large vessels. In BD, an autoinflammatory mechanism is thought to contribute to pathogenesis, and treatment with the TNF-α inhibitor infliximab has been reported to reduce IL-1R2 expression [135]. Furthermore, GWAS of ankylosing spondylitis (AS) have linked *IL1R2* polymorphisms to this disease [136], suggesting that autoinflammatory mechanisms may also be involved in AS pathogenesis [137]. TNF inhibitors are effective not only in BD and AS but also in TAK [138], and the TLR4-mediated expression of IL-1β and IL-1R2 in TAK suggests that, in addition to autoimmunity, autoinflammation may contribute to disease pathogenesis.

The interaction between PD-1 on activated T cells and its ligand PD-L1 on DCs has recently garnered attention in GCA. PD-1 is expressed on the surface of activated T and B cells, and its binding to PD-L1 or PD-L2 inhibits the TCR activation cascade via the phosphatase SHP2. In GCA, vascular DCs have been found to exhibit reduced PD-L1 expression [139]. PD-L1^low^ DCs derived from GCA patients promote T cell activation and proliferation, leading to excessive activation of CD4^+^ T cells, which in turn enhances the production of IFN-γ, IL-17, and IL-21. The accumulation of these multifunctional T cells has been associated with rapid intimal hyperplasia, endothelial activation, and neoangiogenesis, suggesting that T cells contribute to pathological vascular remodeling [139]. Supporting this, multiple case reports have documented that patients receiving immune checkpoint inhibitors are at risk of treatment-induced vasculitis [140]. Restoring PD checkpoint function could serve as a novel therapeutic strategy for medium- and large-sized vessel vasculitis.

In summary, these findings suggest that DCs and their signaling pathways play a crucial role in GCA and other forms of vasculitis.

### Antineutrophil cytoplasmic antibody (ANCA)-associated vasculitis (AAV)

AAV is a chronic, systemic autoimmune disease characterized by inflammation and necrosis of small blood vessels, with rapidly progressing glomerulonephritis and pulmonary hemorrhage being the most severe manifestations. ANCA are autoantibodies that primarily target the neutrophil granule proteins, proteinase 3 (PR3) and myeloperoxidase (MPO). The involvement of specific receptors on DCs recognizing these autoantigens may contribute to the development of autoimmune vasculitis. Interestingly, Csernok et al. demonstrated that PR3 induces DC maturation in vitro, triggering a strong Th1 response, which may promote granuloma formation in granulomatosis with polyangiitis (GPA) [141]. In fact, in GPA patients, cells expressing DC-LAMP (CD208), a marker of mature DCs, have been identified within nasal granulomas [142]. Additionally, in MPO-AAV, the breakdown of tolerance to MPO autoantigens is promoted by TLR9-mediated DC activation [143].

The levels of both circulating cDCs and pDCs in AAV patients were markedly reduced during both the acute and remission phases, with the most pronounced reduction observed in the acute phase [144]. The levels of CD62L, which mediates the migration of pDCs to lymph nodes [145], were increased in both the cDCs and pDCs of AAV patients [144]. Additionally, immature DCs (CD209^+^), presumed to be cDCs, were recruited to the kidneys of AAV patients, where they subsequently formed interstitial infiltrates together with T cells [146]. In GPA patients, pDCs producing type I IFNs were mobilized within pulmonary granulomas, particularly near macrophages and apoptotic neutrophils [147]. These findings suggest that the reduction in DC numbers during the acute phase may be related to the recruitment of these cells to secondary lymphoid tissues and/or inflammatory sites.

Regarding the functional aspects, untreated AAV cases showed a reduction in Treg suppressive activity [144]. Since mature DCs strongly induce Treg proliferation and maintain their suppressive function [148, 149], the impaired Treg function in AAV may be associated with functional alterations in DCs and could represent a critical defect in the tolerogenic checkpoint of AAV. Examining functionality by subset, a significant reduction in the number of circulating pDCs was observed during active AAV, whereas the IFN-α secretion capacity of pDCs in the inflammatory tissues of GPA patients remained intact [147]. On the contrary, in cDCs from the peripheral blood of GPA and MPA patients, IL-12/IL-23p40 production in response to multiple TLR ligands was reduced, which may contribute to abnormal Th cell responses [144]. However, the cytokine production capacity of cDCs infiltrating AAV-affected tissues has not been evaluated. Further investigation is needed to assess the antigen-presenting and T cell-stimulating capacities of DCs.

## Conclusions

This review highlights that DCs are involved to varying degrees in different connective tissue diseases and abnormalities in activation or interactions with other cells contribute to inflammation.

The first key point regarding the role of DCs in connective tissue diseases is their localization. In conditions such as SSc, SLE, and AAV, pDC numbers are reduced in peripheral blood, possibly due to the migration of pDCs into tissues. However, changes in DC numbers or localization alone are unlikely to be the direct cause of autoimmune diseases. Instead, as the second key point, functional alterations in DCs may trigger inflammation and autoimmunity.

In the diseases reviewed, DC migration to inflammatory sites has been observed; however, the DC subsets primarily contributing to pathogenesis vary among the diseases. pDCs appear to play a major role in SLE and SSc, whereas cDCs may be more involved in RA (Table 1). Although type I IFN produced by pDCs is a common pathogenic factor in both SLE and SSc, it contributes to inflammation in SLE versus fibrosis in SSc. On the contrary, the strong antigen-presenting ability of cDCs is a shared mechanism across multiple diseases, as it facilitates the priming and/or differentiation of autoreactive T cells, leading to inappropriate activation signals and breakdown of intrinsic regulatory mechanisms. In RA, disease progression may involve functional changes in the cDC1s and cDC2s migrating into the synovium, along with unique subsets such as inflammatory DC3s and LAMP3^+^ DCs, which potentially differentiated from DC2s. In vasculitis, the presence of vessel wall-residing vascular DCs is particularly intriguing. While certain signaling pathways in DCs are important, the specific DC subsets constituting vascular DCs and their functional roles remain insufficiently characterized. In IIM, DC accumulation in muscle tissue is thought to contribute to sustained immune-mediated reactions and further muscle damage. However, differences in the pathogenic mechanisms among DC subsets complicate disease understanding. Among the diseases reviewed, IIM remains one of the least understood in terms of DC involvement, warranting further investigation. Since 2017, advances in the identification of DC subsets have revealed their heterogeneity and functional diversity. However, functional studies of these newly identified subsets in diseases other than RA and SLE are lacking, highlighting the need for further disease-specific research. CTD-PAH could be a key area for cross-disease research of DCs, but studies on DC involvement in CTD-PAH are currently limited.

**Table 1:** A summary of the changes in DCs and their impact on each disease

	In peripheral blood		In tissues	
	cDCs	pDCs	cDCs	pDCs
**RA**	(controversial)	↓ (controversial)	**・LAMP3**^**+**^ **DCs and iDC3s are present in the synovium and co-localize with T cells.** ・The migration of cDCs from peripheral blood to the synovium and their functional changes may be associated with disease pathogenesis and activity.	
**SLE**	Pro-inflammatory phenotype/function	↓	Interactions between activated cDCs and other cells may contribute to the disease process.	**Excessive activation of pDCs and overproduction of type I IFN play a central role in the pathogenesis.**
**IIM**			Dominate in PM and IBM	Dominate in DM
			**The accumulation of DCs in muscle tissue may contribute to the progression of persistent immune-mediated reactions and further muscle damage.**	
**SSc**		↓		**↑ (in the skin and lungs)** **pDCs may be responsible for fibrosis through type I IFN and CXCL4.**
**Vasculitis**			**・The vessel wall-specific “vascular DCs” and their signaling pathways play an important role.** ・The TLR profile expressed by vascular DCs is specific to the type of vessel, which may explain the different affected vessels in each vasculitis.	
**AAV**	↓ (especially during the acute phase)	↓ (especially during the acute phase)	The migration of DCs to the site of inflammation and the functional decline of cDCs may contribute, but further evaluation is needed in the future.	

*RA* rheumatoid arthritis, *SLE* systemic lupus erythematosus, *IIM* idiopathic inflammatory myopathy, *SSc* systemic sclerosis, *AAV* ANCA-associated vasculitis, *DC*, dendritic cell, *cDC* conventional dendritic cells, *pDC* plasmacytoid dendritic cells, *DM* dermatomyositis, *PM* polymyositis, *IBM* inclusion body myositis, *iDC* inflammatory DC, *IFN* interferon, *TLR* Toll-like receptor

Aberrant DC activation contributes to autoimmune manifestations. A deeper understanding of DC involvement in individual autoimmune diseases could lead to therapeutic interventions targeting common DC-mediated mechanisms across multiple diseases.

## Data Availability

Not applicable.

## Abbreviations

AAV: Antineutrophil cytoplasmic antibody-associated vasculitis
AMP program: Accelerating Medicines Partnership program
AS: Ankylosing spondylitis
BD: Behçet’s disease
Bregs: Regulatory B cells
cDCs: Conventional dendritic cells
CTD: Connective tissue disease
CXCL4: C–X–C motif ligand 4
DCs: Dendritic cells
dcSSc: Diffuse cutaneous SSc
DM: Dermatomyositis
dsDNA: Double-stranded DNA
FDCs: Follicular dendritic cells
GC: Germinal center
GCA: Giant cell arteritis
GPA: Granulomatosis with polyangiitis
GWAS: Genome-Wide Association studies
HLA: Human leukocyte antigen
IBM: Inclusion body myositis
iDC3: Inflammatory DC3
IFN: Interferon
IFNAR: Type I IFN receptor
IIM: Idiopathic inflammatory myopathy
IL: Interleukin
ILD: Interstitial lung disease
IPAH: Idiopathic PAH
lcSSc: Limited cutaneous SSc
LN: Lupus nephritis
MAC: Membrane attack complex
moDCs: Monocyte-derived dendritic cells
MPO: Myeloperoxidase
NETs: Neutrophil extracellular traps
PAH: Pulmonary arterial hypertension
pDCs: Plasmacytoid dendritic cells
PDGF: Platelet-derived growth factor
PM: Polymyositis
PR3: Proteinase 3
pre-DCs: CDC precursors
RA: Heumatoid arthritis
scRNA-seq: Single-cell RNA sequencing
SLE: Systemic lupus erythematosus
SSc: Systemic sclerosis
TAK: Takayasu arteritis
Tem cells: Wffector memory T cells
TLOs: Tertiary lymphoid organs
TLR: Toll-like receptor
TNF: Tumor necrosis factor
Tph cells: T peripheral helper cells
Tregs: Regulatory T cells
